# Pleural effusion with rib fractures in infant

**DOI:** 10.1002/ccr3.1520

**Published:** 2018-04-10

**Authors:** Akinobu Taniguchi, Takashi Maeda, Takashi Tachibana

**Affiliations:** ^1^ Department of Neonatology Ogaki Municipal Hospital 4‐86 Minaminokawacho Ogaki 503‐8502 Japan

**Keywords:** Child abuse, infant, pleural effusion, rib fracture, shaken baby syndrome

## Abstract

The causes of pleural effusions in children are various. This case demonstrates the importance of considering rib fractures associated with child abuse in the differential diagnosis of pleural effusion in infants.

A 5‐month‐old girl presented with breathing difficulty and poor feeding. On admission, she had a fever of 38°C, cyanosis, 85% oxygen saturation on pulse oximetry, and lethargy. Physical examination revealed decreased breath sounds bilaterally. There were not heart murmur, skin bruising, and neurological signs. Chest radiography revealed bilateral pleural fluid (Fig. 1). Chest computed tomography revealed bilateral pleural fluid and multiple posterolateral and posteromedial rib fractures (Figs. 2 and 3: arrows indicate fractures). Aspirated pleural fluid was exudative and nonhemorrhagic. We performed X‐ray of limbs and computed tomography of head and neck, but no other fracture was identified. As there was no other history of injury, bilateral pleural fluid with multiple rib fractures was considered the result of shaken baby syndrome. There was no family history, and we found no underlying disease. The pleural effusion resolved spontaneously, and the rib fractures healed without intervention.

**Figure 1:**
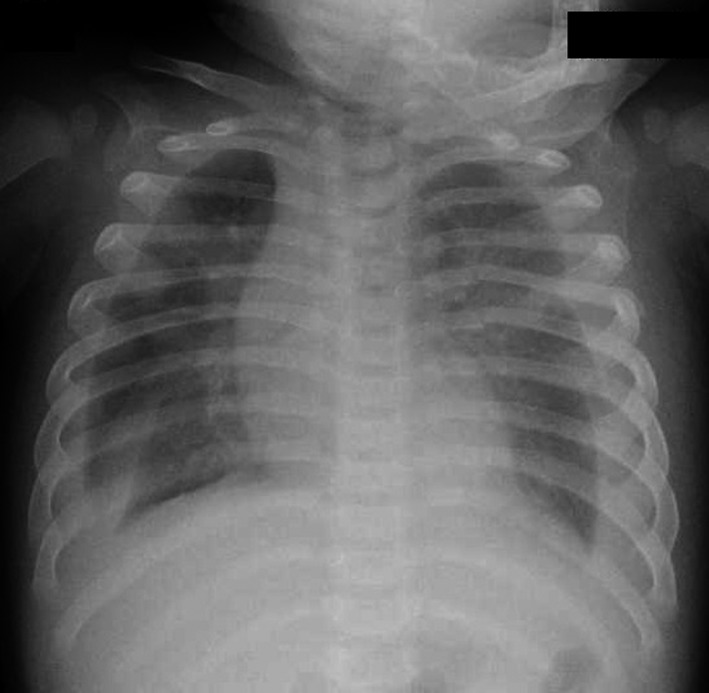
Chest radiography shows bilateral pleural fluid.

**Figure 2:**
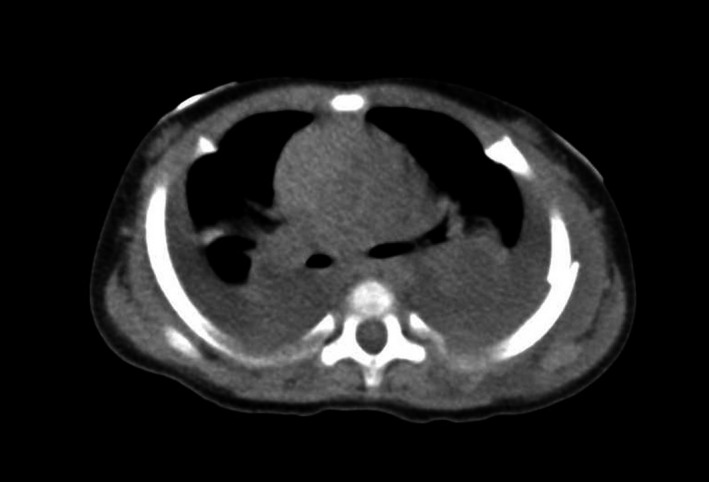
Chest computed tomography shows bilateral pleural fluid and rib fracture.

**Figure 3:**
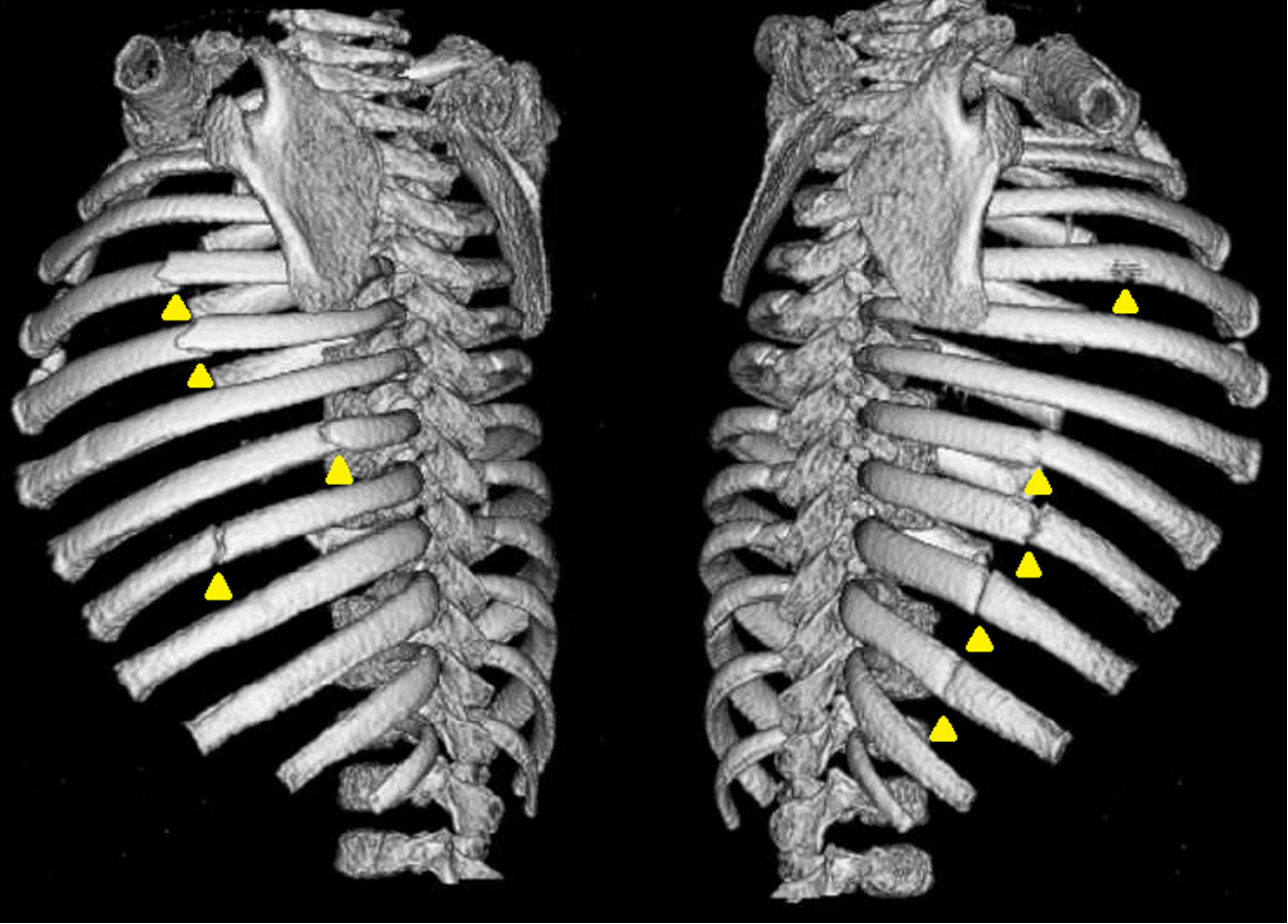
3D chest computed tomography of bone shows multiple posterolateral and posteromedial rib fractures.

Multiple posterior rib fractures in infants are classically associated with nonaccidental trauma 1. It occurs when a baby is severely shaken while being held by the chest. Nonhemorrhagic pleural effusion after rib fractures in adults is thought to be due to pleural irritation by broken ribs 2; however, such report is rare in infant.

## Conflict of Interest

No potential conflict of interest to disclose.

## Informed Consent

The signed consent was obtained from caregiver (mother) in writing.

## Authorship

AT and TM: contributed to this report as a physician in charge of the treatment of this case. AT: drafted this report. TT: assisted in the preparation of the manuscript.
